# Supplementary material for: From sequence to enzyme mechanism using multi-label machine learning
Source: BMC Bioinformatics. 2014 May 19;15:150. doi: 10.1186/1471-2105-15-150 (PMC4229970; doi:10.1186/1471-2105-15-150)
Supplement: Additional file 2 — Java code of ml2db. Additional file ml2db_code.tar.gz contains the Java source code to run the multi-label machine learning experiments and save the results to database. The code’s Javadoc is included. [file 1471-2105-15-150-S2.zip › additional file 2/ml2db/utils/doc/uk/ac/standrews/utils/test/maputils/TableMapTest.html]

TableMapTest


JavaScript is disabled on your browser.


- Overview
- Package
- Class
- Use
- Tree
- Deprecated
- Index
- Help

- Prev Class
- Next Class

- Frames
- No Frames

- All Classes

- Summary:
- Nested |
- Field |
- Constr |
- Method

- Detail:
- Field |
- Constr |
- Method


uk.ac.standrews.utils.test.maputils

## Class TableMapTest

- java.lang.Object
- - junit.framework.Assert
  - - junit.framework.TestCase
    - - uk.ac.standrews.utils.test.maputils.TableMapTest

- All Implemented Interfaces:
  :   junit.framework.Test

  ---

    

  ```
  public class TableMapTest
  extends junit.framework.TestCase
  ```

  Class

  Version:
  :   28 May 2008

  Author:
  :   Luna De Ferrari luna.deferrari-at-ed.ac.uk

- - ### Constructor Summary

    Constructors

    Constructor and Description

    `TableMapTest()`
  - ### Method Summary

    Methods

    | Modifier and Type | Method and Description |
    | --- | --- |
    | `static TableMap` | `map()` |
    | `void` | `testAddValue()` |
    | `void` | `testFillMap()` |
    | `void` | `testGetKeys()` |
    | `void` | `testGetKeyValueNumber()` |
    | `void` | `testGetValues()` |
    | `void` | `testGetValuesForAKey()` |
    | `void` | `testRemoveValue()` |

    - ### Methods inherited from class junit.framework.TestCase

      `countTestCases, getName, run, run, runBare, setName, toString`
    - ### Methods inherited from class junit.framework.Assert

      `assertEquals, assertEquals, assertEquals, assertEquals, assertEquals, assertEquals, assertEquals, assertEquals, assertEquals, assertEquals, assertEquals, assertEquals, assertEquals, assertEquals, assertEquals, assertEquals, assertEquals, assertEquals, assertEquals, assertEquals, assertFalse, assertFalse, assertNotNull, assertNotNull, assertNotSame, assertNotSame, assertNull, assertNull, assertSame, assertSame, assertTrue, assertTrue, fail, fail, failNotEquals, failNotSame, failSame, format`
    - ### Methods inherited from class java.lang.Object

      `equals, getClass, hashCode, notify, notifyAll, wait, wait, wait`

- - ### Constructor Detail


    - #### TableMapTest

      ```
      public TableMapTest()
      ```
  - ### Method Detail


    - #### map

      ```
      public static TableMap map()
      ```


    - #### testAddValue

      ```
      public void testAddValue()
      ```


    - #### testFillMap

      ```
      public void testFillMap()
      ```


    - #### testGetKeys

      ```
      public void testGetKeys()
      ```


    - #### testGetKeyValueNumber

      ```
      public void testGetKeyValueNumber()
      ```


    - #### testGetValues

      ```
      public void testGetValues()
      ```


    - #### testGetValuesForAKey

      ```
      public void testGetValuesForAKey()
      ```


    - #### testRemoveValue

      ```
      public void testRemoveValue()
      ```


- Overview
- Package
- Class
- Use
- Tree
- Deprecated
- Index
- Help

- Prev Class
- Next Class

- Frames
- No Frames

- All Classes

- Summary:
- Nested |
- Field |
- Constr |
- Method

- Detail:
- Field |
- Constr |
- Method
